# A Powerful Method To Test Associations Between Ordinal Traits and Genotypes

**DOI:** 10.1534/g3.119.400293

**Published:** 2019-06-05

**Authors:** Jinjuan Wang, Juan Ding, Shouyou Huang, Qizhai Li, Dongdong Pan

**Affiliations:** * LSC, NCMIS, Academy of Mathematics and Systems Science, Chinese Academy of Sciences, Beijing, 100190, China,; † University of Chinese Academy of Sciences, Beijing, 100049, China,; ‡ School of Mathematics and Statistics, Guangxi Normal University, Guilin, 541004, China,; § School of Mathematics and Statistics, Hubei Normal University, Huangshi, 435002, China,; ** Yunnan Key Laboratory of Statistical Modeling and Data Analysis, Yunnan University, Kunming, 650500, China, and; †† Department of Biostatistics, School of Public Health, Yale University, New Haven, CT 06511

**Keywords:** association study, ordinal phenotype, latent normal variate, generalized estimating equation, M-estimation

## Abstract

The methods commonly used to test the associations between ordinal phenotypes and genotypes often treat either the ordinal phenotype or the genotype as continuous variables. To address limitations of these approaches, we propose a model where both the ordinal phenotype and the genotype are viewed as manifestations of an underlying multivariate normal random variable. The proposed method allows modeling the ordinal phenotype, the genotype and covariates jointly. We employ the generalized estimating equation technique and M-estimation theory to estimate the model parameters and deduce the corresponding asymptotic distribution. Numerical simulations and real data applications are also conducted to compare the performance of the proposed method with those of methods based on the logit and probit models. Even though there may be potential limitations in Type I error rate control for our method, the gains in power can prove its practical value in case of exactly ordinal phenotypes.

Research in the field of genetic epidemiology suggests that some genetic variants play important roles in the etiology of human diseases. On the one hand, genetic variants are defined as genotypes, which are often treated as ordinal variables. On the other hand, there are multiple data types for diseases, *i.e.*, phenotypes, which can be continuous, binary, or ordinal (Li *et al.* 2006; Kim *et al.* 2013). Note that both binary and ordinal variables are categorical variables, but the latter can describe the disease state of a patient more precisely in many circumstances. For example, four levels—normal liver, light steatosis, moderate steatosis, and severe steatosis—have been utilized to describe the severity of liver steatosis (Bedogni *et al.* 2010).

With the development of high throughput biologic technology, increasingly more genotypes and data with complex traits have been generated and deposited in public databases. It is urgently required to develop new statistical testing methods to investigate the associations between these and extract useful information to understand the underlying occurrence and development mechanisms of diseases and traits.

Genome-wide association studies aim to identify associations between phenotypes and genotypes. In these studies, genotypes are often treated as predictors and phenotypes as outcomes. If the phenotype of interest is continuous, then the classic linear regression model is commonly employed. When the phenotype is ordinal, the multinomial logit model (McCullagh 1980; Zhang *et al.* 2015) or ordered probit model (Daykin and Moffatt 2002; Wang 2014) should be recommended. All these models regress phenotype values or their distribution-based transformations on genotypes, with the assumptions that genotype values are continuous (Korse *et al.* 2009; Bedogni *et al.* 2010) and the probability of having a disease increases linearly with the genotype value. However, the continuity assumption on genotype values and the linearity assumption between a phenotype and genotype are difficult to verify in practice. If these two assumptions are violated, the corresponding Wald testing statistics may severely decrease in power. To overcome this, some researchers treated genotypes as ordinal variables and reversed the regression process by regressing genotypes on phenotypes (O’Reilly *et al.* 2012). When a phenotype is a continuous variable, this new method is indeed useful for removing or relaxing the continuity and linearity assumptions. However, this does not work when a phenotype is exactly ordinal, such as in the above-mentioned example of liver steatosis. Therefore, we propose a new method to deal with this problem.

In this work, we treat genotypes as ordinal variables and propose a new procedure to assess the association between an ordinal phenotype and ordinal genotype after adjusting for covariates. Rather than regressing the phenotype on the genotype or regressing the genotype on the phenotype using existing methods, we jointly model the phenotype and genotype by introducing a latent variable following a multivariate normal distribution. The phenotype and genotype are regarded as manifestation values of the latent variable. The relationships between phenotypes, genotypes, and covariates of interest are elaborately described by the covariance matrix. Taking advantage of the framework of generalized estimation equations (Hanley *et al.* 2003; Zhang *et al.* 2014) and M-estimation theory (Huber 1981; Stefanski and Boos 2002), we construct a Wald test statistic for an equivalent transformation of the original null hypothesis, and prove that it asymptotically follows the standard normal distribution under the null hypothesis. Numerical simulations are conducted to compare the proposed method with other methods. Our simulation results show that the proposed method can suitably maintain Type I error control and may achieve considerable statistical power compared to existing methods in various scenarios. Finally, we apply the proposed method to anticyclic citrullinated protein antibody data for rheumatoid arthritis studies, to further demonstrate its performance.

## Materials And Methods

### Notations

Let random variables *G* and *Y* denote a genotype and ordinal phenotype, respectively, and $Z={\left(Z_{1},Z_{2},\cdots ,Z_{k-2}\right)}^{\tau }$ be a (k−2)-dimensional continuous covariate of interest, with k≥3, where *τ* denotes the transpose of a matrix or vector. Without loss of generality, we assume that there are two alleles at a genetic locus, with one being the risk allele and the other being the reference allele. The value of the random variable *G* represents the number of the risk allele at a locus, which means that *G* can take three values: 0, 1, or 2. Suppose that *Y* takes *m* ordinal values: 1,2,⋯,m. The null hypothesis states that after adjusting for covariates, the phenotype is not related to the genotype, *i.e.*, the phenotype and genotype are conditionally independent given a set of covariates, which can be denoted by

$$H_{01}:\text{Pr}\left(G,Y|Z\right)=\text{Pr}\left(G|Z\right)\text{Pr}\left(Y|Z\right).$$ (1)

Assume that *n* subjects are enrolled in the genetic association study. Further, let $\left\{g_{1},g_{2},\cdots ,g_{n}\right\}$, $\left\{y_{1},y_{2},\cdots ,y_{n}\right\}$, and $\left\{z_{1},z_{2},\cdots ,z_{n}\right\}$ be the *n* observations of *G*, *Y*, and Z corresponding to the *n* subjects, respectively.

### Equivalent statement of $H_{\mathrm{01}}$ by introducing a latent variable U

We assume that the combined vector ${\left(G,Y,Z^{\tau }\right)}^{\tau }$ is generated from a *k*-dimensional random variable $U={\left(U_{1},U_{2},\cdots ,U_{k}\right)}^{\tau }$ following a multivariate normal distribution, with mean vector $0_{k}={\left(0,0,\cdots ,0\right)}_{k\times 1}^{\tau }$ (the *k*-dimensional column vector with all units being zero) and covariance matrix Δ, where $\text{Δ}={\left(\delta_{k_{1}k_{2}}\right)}_{k\times k}$ and all its diagonal elements are equal to one. That is, (G,Y) represents the manifestation of $U_{01}=\left(U_{1},U_{2}\right)$ and $Z={\left(U_{3},\cdots ,U_{k}\right)}^{\tau }$. We rewrite Δ in the partitioned matrix form

$$\text{Δ}=\left(\begin{array}{cc} {\text{Δ}}_{11} & {\text{Δ}}_{12}\\ {\text{Δ}}_{21} & {\text{Δ}}_{22} \end{array}\right),$$ (2)

where ${\text{Δ}}_{11}$ is a 2 × 2 matrix. Then, Z follows a multivariate normal distribution with mean vector $0_{k-2}={\left(0,0,\cdots ,0\right)}_{\left(k-2\right)\times 1}^{\tau }$ and covariance matrix ${\text{Δ}}_{22}$. Then, *G* and *Y* can be obtained as follows:

$$G=\{\begin{array}{cc} 0, & -\infty <U_{1}\leq \xi_{1};\\ 1, & \xi_{1}<U_{1}\leq \xi_{2};\\ 2, & \xi_{2}<U_{1}<+\infty ; \end{array}$$ (3)

and

$$Y=\{\begin{array}{cc} 1, & -\infty <U_{2}\leq \eta_{1};\\ 2, & \eta_{1}<U_{2}\leq \eta_{2};\\ \ldots & \\ m, & \eta_{m-1}<U_{2}<+\infty , \end{array}$$ (4)

where $-\infty <\xi_{1}<\xi_{2}<+\infty$ and $-\infty <\eta_{1}<\eta_{2}<\cdots <\eta_{m-1}<+\infty$.

Based on the theory of the conditional normal distribution, we have that

$${\left(U_{1},U_{2}\right)}^{\tau }|Z∼N\left({\text{Δ}}_{12}{\text{Δ}}_{22}^{-1}Z,{\text{Δ}}_{11}-{\text{Δ}}_{12}{\text{Δ}}_{22}^{-1}{\text{Δ}}_{21}\right).$$ (5)

We define $\zeta_{1}={\left(\delta_{13},\delta_{14},\cdots ,\delta_{1k}\right)}^{\tau },\zeta_{2}={\left(\delta_{23},\delta_{24}\cdots ,\delta_{2k}\right)}^{\tau }.$ Then, the conditional covariance matrix above can be further expressed as

$$\begin{array}{l} {\text{Δ}}_{11}-{\text{Δ}}_{12}{\text{Δ}}_{22}^{-1}{\text{Δ}}_{21}=\left(\begin{array}{cc} 1 & \delta_{12}\\ \delta_{12} & 1 \end{array}\right)-\left(\begin{array}{c} \zeta_{1}^{\tau }\\ \zeta_{2}^{\tau } \end{array}\right){\text{Δ}}_{22}^{-1}\left(\zeta_{1},\zeta_{2}\right)\\ \;=\left(\begin{array}{cc} 1-\zeta_{1}^{\tau }{\text{Δ}}_{22}^{-1}\zeta_{1} & \delta_{12}-\zeta_{1}^{\tau }{\text{Δ}}_{22}^{-1}\zeta_{2}\\ \delta_{12}-\zeta_{2}^{\tau }{\text{Δ}}_{22}^{-1}\zeta_{1} & 1-\zeta_{2}^{\tau }{\text{Δ}}_{22}^{-1}\zeta_{2} \end{array}\right). \end{array}$$ (6)

Now, denote $\delta_{0}={\left(\delta_{12},\zeta_{1}^{\tau },\zeta_{2}^{\tau }\right)}^{\tau }$, $\varrho \left(\delta_{0}\right)=\delta_{12}-\zeta_{1}^{\tau }{\text{Δ}}_{22}^{-1}\zeta_{2}$. By introducing the latent variable U and taking advantage of its distribution property, we can state that the original hypothesis $H_{01}$ is exactly equivalent to

$$H_{02}:\varrho \left(\delta_{0}\right)=0.$$ (7)

### Proposed statistical test

In this subsection, we construct a test statistic to test $H_{02}$ based on the generalized estimating equation technique and M-estimation theory. Recall that the joint distribution of $U_{1}$ and $Z^{\tau }$ is

$${\left(U_{1},Z^{\tau }\right)}^{\tau }∼N\left(0_{k-1},\left(\begin{array}{cc} 1 & \zeta_{1}^{\tau }\\ \zeta_{1} & {\text{Δ}}_{22} \end{array}\right)\right).$$ (8)

Hence, the conditional distribution of $U_{1}$ given Z is

$$U_{1}|Z∼N\left(\zeta_{1}^{\tau }{\text{Δ}}_{22}^{-1}Z,1-\zeta_{1}^{\tau }{\text{Δ}}_{22}^{-1}\zeta_{1}\right).$$ (9)

Similarly, the conditional distribution of $U_{2}$ given Z is

$$U_{2}|Z∼N\left(\zeta_{2}^{\tau }{\text{Δ}}_{22}^{-1}Z,1-\zeta_{2}^{\tau }{\text{Δ}}_{22}^{-1}\zeta_{2}\right).$$ (10)

Denote $\xi_{0}=-\infty ,\xi_{3}=+\infty ,\eta_{0}=-\infty ,\eta_{m}=+\infty$. It should be noted that the marginal density function of Z and the joint density functions of each pair of variables among G,Y, and Z are as follows:

$$f_{Z}\left(z;{\text{Δ}}_{22}\right)=\frac{1}{{\left(2\pi \right)}^{\frac{k-2}{2}}{\left|{\text{Δ}}_{22}\right|}^{\frac{1}{2}}}\text{exp}\left(-\frac{z^{\tau }{\text{Δ}}_{22}^{-1}z}{2}\right)$$ (11)

$$\begin{array}{l} f_{G,Y}\left(g,y;\delta_{12}\right)=\underset{\begin{array}{c} 1\leq j\leq 3\\ 1\leq r\leq m \end{array}}{\prod }[\int_{\eta_{r-1}}^{\eta_{r}}\int_{\xi_{j-1}}^{\xi_{j}}\frac{1}{2\pi \sqrt{1-\delta_{12}^{2}}}\\ \;{\text{exp}\left(-\frac{u_{1}^{2}-2\delta_{12}u_{1}u_{2}+u_{2}^{2}}{2\left(1-\delta_{12}^{2}\right)}\right)\text{d}u_{1}\text{d}u_{2}]}^{\text{I}\left(g=j,y=r\right)} \end{array}$$ (12)

$$\begin{array}{l} f_{G,Z}\left(g,z;\zeta_{1}\right)=f_{Z}\left(z;{\text{Δ}}_{22}\right)\overset{3}{\underset{j=1}{\prod }}[\int_{\xi_{j-1}}^{\xi_{j}}\frac{1}{\sqrt{2\pi \left(1-\zeta_{1}^{\tau }{\text{Δ}}_{22}^{-1}\zeta_{1}\right)}}\\ \;{\text{exp}\left(-\frac{{\left(u_{1}-\zeta_{1}^{\tau }{\text{Δ}}_{22}^{-1}z\right)}^{2}}{2\left(1-\zeta_{1}^{\tau }{\text{Δ}}_{22}^{-1}\zeta_{1}\right)}\right)\text{d}u_{1}]}^{\text{I}\left(g=j\right)} \end{array}$$ (13)

$$\begin{array}{l} f_{Y,Z}\left(y,z;\zeta_{2}\right)=f_{Z}\left(z;{\text{Δ}}_{22}\right)\overset{m}{\underset{r=1}{\prod }}[\int_{\eta_{r-1}}^{\eta_{r}}\frac{1}{\sqrt{2\pi \left(1-\zeta_{2}^{\tau }{\text{Δ}}_{22}^{-1}\zeta_{2}\right)}}\\ \;{\text{exp}\left(-\frac{{\left(u_{2}-\zeta_{2}^{\tau }{\text{Δ}}_{22}^{-1}z\right)}^{2}}{2\left(1-\zeta_{2}^{\tau }{\text{Δ}}_{22}^{-1}\zeta_{2}\right)}\right)\text{d}u_{2}]}^{\text{I}\left(y=r\right)} \end{array}$$ (14)

where I(E) is an indicator function, *i.e.*, I(E) is one if the event *E* holds and zero otherwise. The unknown parameters ${\text{Δ}}_{22},\left\{\xi_{j},j=1,2\right\},\left\{\eta_{r},r=1,\cdots ,m-1\right\}$ and $\delta_{0}$ can be estimated via the following procedures.

First, based on the marginal density function (11) of Z, we have the likelihood function

$$L\left({\text{Δ}}_{22}\right)=\overset{n}{\underset{i=1}{\prod }}f_{Z}\left(z_{i};{\text{Δ}}_{22}\right)=\overset{n}{\underset{i=1}{\prod }}\frac{1}{{\left(2\pi \right)}^{\frac{k-2}{2}}{\left|{\text{Δ}}_{22}\right|}^{\frac{1}{2}}}\text{exp}\left(-\frac{z_{i}^{\tau }{\text{Δ}}_{22}^{-1}z_{i}}{2}\right).$$ (15)

By maximizing $L\left({\text{Δ}}_{22}\right)$ on ${\text{Δ}}_{22}$, we can obtain the MLE of ${\text{Δ}}_{22}$ (denoted by Δ^22), which is the sample covariance matrix of the observed data $\left\{z_{1},z_{2},\cdots ,z_{n}\right\}$.

Second, in our model both *G* and *Y* are ordinal variables, whose realizations are determined by the intervals in which the values of two standard normal random variables $U_{1}$ and $U_{2}$ may fall in, respectively. Specifically, we employ the distribution properties of *G* and *Y* to intuitively estimate $\left\{\xi_{j},j=1,2\right\}$ and $\left\{\eta_{r},r=1,\cdots ,m-1\right\}$, respectively. We define $\lambda_{1}=\frac{\#\{g_{i}=0,i=1,2\cdots ,n\}}{n}$, $\lambda_{2}=\frac{\#\{g_{i}=1,i=1,2\cdots ,n\}}{n}$, $\lambda_{3}=\frac{\#\{g_{i}=2,i=1,2\cdots ,n\}}{n}$, and $\omega_{r}=\frac{\#\{y_{i}=r,i=1,2\cdots ,n\}}{n}$ for r=1,2,⋯,m based on the observed data $\left\{g_{1},g_{2},\cdots ,g_{n}\right\}$ and $\left\{y_{1},y_{2},\cdots ,y_{n}\right\}$ of *G* and *Y*, respectively. Recall that $\xi_{0}=\eta_{0}=-\infty$. Then, we can estimate $\xi_{j}$ as ${\hat{\xi }}_{j}$, j=1,2 and $\eta_{r}$ as ${\hat{\eta }}_{r}$, r=1,⋯,m−1 by solving the following equations:

$$\lambda_{j}=\text{Φ}\left(\xi_{j}\right)-\text{Φ}\left(\xi_{j-1}\right),j=1,2;$$ (16)

$$\omega_{r}=\text{Φ}\left(\eta_{r}\right)-\text{Φ}\left(\eta_{r-1}\right),r=1,\cdots ,m;$$ (17)

where Φ(⋅) is the cumulative distribution function of the standard normal variable.

The parameter $\delta_{0}$ can be estimated using the generalized estimating equation technique and M-estimation theory. First, let

$$\begin{array}{l} h\left(\delta_{0}\right)=\sum_{i=1}^{n}\text{log}\;f_{G,Y}\left(g_{i},y_{i};\delta_{12}\right)\\ \;+\sum_{i=1}^{n}\text{log}\;f_{G,Z}\left(g_{i},z_{i};\zeta_{1}\right)+\sum_{i=1}^{n}\text{log}\;f_{Y,Z}\left(y_{i},z_{i};\zeta_{2}\right). \end{array}$$ (18)

The function vector consisting of the first-order partial derivatives of $h\left(\delta_{0}\right)$ with respect to each parameter in $\delta_{0}$ is

$$\begin{array}{l} \phi \left(G,Y,Z;\delta_{0}\right)=[\frac{\partial \text{log}\;f_{G,Y}\left(G,Y;\delta_{12}\right)}{\partial \delta_{12}},\\ \;{{\left(\frac{\partial \text{log}\;f_{G,Z}\left(G,Z;\zeta_{1}\right)}{\partial \zeta_{1}}\right)}^{\tau },{\left(\frac{\partial \text{log}\;f_{Y,Z}\left(Y,Z;\zeta_{2}\right)}{\partial \zeta_{2}}\right)}^{\tau }]}^{\tau }. \end{array}$$ (19)

Then, the estimator ${\hat{\delta }}_{0}={\left({\hat{\delta }}_{12},{\hat{\zeta }}_{1}^{\tau },{\hat{\zeta }}_{2}^{\tau }\right)}^{\tau }$ of $\delta_{0}$ is the root of the following generalized estimating equation

$$\sum_{i=1}^{n}\phi \left(g_{i},y_{i},z_{i};\delta_{0}\right)=0.$$ (20)

After estimating all the unknown parameters, the estimate of $\varrho \left(\delta_{0}\right)$ can be expressed as ϱ(δ^0)=δ^12−ζ^1τΔ^22−1ζ^2. To construct a Wald-type test statistic, we need to derive the asymptotical distribution of $\varrho \left({\hat{\delta }}_{0}\right)$. Based on the classical M-estimation theory, ${\hat{\delta }}_{0}$ asymptotically follows a multivariate normal distribution. That is,

$$\sqrt{n}\left({\hat{\delta }}_{0}-\delta_{0}\right)\;\overset{{}^{dist.}}{\to }\;N\left(0_{2k-3},V\left(\delta_{0}\right)\right)\;\text{as}\;n\to \infty ,$$ (21)

where $V\left(\delta_{0}\right)=J{\left(\delta_{0}\right)}^{-1}I\left(\delta_{0}\right)J{\left(\delta_{0}\right)}^{-1}$, $I\left(\delta_{0}\right)=\frac{1}{n}\sum_{i=1}^{n}\phi \left(g_{i},y_{i},z_{i};\delta_{0}\right)\phi^{\tau }\left(g_{i},y_{i},z_{i};\delta_{0}\right)$, and $J\left(\delta_{0}\right)=-\frac{1}{n}\sum_{i=1}^{n}\frac{\partial \phi \left(g_{i},y_{i},z_{i};\delta_{0}\right)}{\partial \delta_{0}^{\tau }}.$

According to the delta method, $\varrho \left({\hat{\delta }}_{0}\right)$ is also asymptotically multivariate normal. Namely,

$$\sqrt{n}\left(\varrho \left({\hat{\delta }}_{0}\right)-\varrho \left(\delta_{0}\right)\right)\;\overset{{}^{dist.}}{\to }N\left(0,{\left(\overset{\cdot }{\varrho }\left(\delta_{0}\right)\right)}^{\tau }V\left(\delta_{0}\right)\overset{\cdot }{\varrho }\left(\delta_{0}\right)\right)\;\text{as}\;n\to \infty ,$$ (22)

where $\overset{\cdot }{\varrho }\left(\delta_{0}\right)=\frac{\partial \varrho \left(\delta_{0}\right)}{\partial \delta_{0}}={\left(1,-{\left({\text{Δ}}_{22}^{-1}\zeta_{2}\right)}^{\tau },-\zeta_{1}^{\tau }{\text{Δ}}_{22}^{-1}\right)}^{\tau }$.

Now, we can propose a new Wald test statistic for the null hypothesis $H_{02}$ as follows:

$$T_{W}=\frac{\varrho \left({\hat{\delta }}_{0}\right)}{\sqrt{{\left(\overset{\cdot }{\varrho }\left({\hat{\delta }}_{0}\right)\right)}^{\tau }V\left({\hat{\delta }}_{0}\right)\overset{\cdot }{\varrho }\left({\hat{\delta }}_{0}\right)/n}}\;\overset{{}^{dist.}}{\to }N\left(0,1\right)\;\text{as}\;n\to \infty .$$ (23)

### Data availability

The authors affirm that all data necessary for confirming the conclusions of the article are present within the article, figures, and tables. Supplemental material available at FigShare: https://doi.org/10.25387/g3.8226650.

## Results

### Simulation results

In this subsection, we present a series of simulation studies to investigate the performance of our proposed latent variable model (abbreviated as lvm), and compare it with the probit and logit models, which both regressing an ordinal phenotype on a genotype. We compared these under multiple simulation scenarios, so that different modeling assumptions would be favored. Two types of data generation mechanism were considered throughout our simulation studies: (i) generating data from a multivariate normal random variable (named the ND mechanism) and (ii) generating data under the proportional odds model (named the PO mechanism). In addition, three genetic models (co-dominant, dominant, and recessive models) were considered.

The specifics of our simulation data generation scenarios are as follows. For simplicity, the dimension of the covariate Z is one, and the number of levels for a phenotype *Y* is set to five. Under the ND mechanism, the three-dimensional latent variable U was generated from a multivariate normal distribution, with the genotype *G* being a manifestation of $U_{1}$, the ordinal phenotype *Y* being a manifestation of $U_{2}$, and the covariate Z being equal to $U_{3}$. Each marginal distribution of $U_{1},U_{2},\text{ and }U_{3}$ followed a standard normal distribution. Note that the distribution of *G* varied according to the type of the true genetic model. Let *A* (major allele) and *a* (minor allele) denote two alleles at the single biallelic locus corresponding to the genotype *G*. Under the Hardy–Weinberg equilibrium (HWE) conditions, the expected genotype frequencies of *G* being AA, Aa, and aa would be ${\left(1-p\right)}^{2},2p\left(1-p\right)$, and $p^{2}$, respectively, with the minor allele frequency (MAF) *p* taking on values from {0.1,0.2,0.3,0.4,0.5}. If the HWE assumption did not hold, we directly set three kinds of multinomial distribution (P) for (AA, Aa, aa) with different parameter structures ${\text{P}}_{1}=\left(0.2,0.3,0.5\right),{\text{P}}_{2}=\left(0.2,0.5,0.3\right)$, and ${\text{P}}_{3}=\left(0.2,0.7,0.1\right)$. When a co-dominant model was assumed, the three genotypes AA, Aa, and aa were coded as 0, 1, and 2, respectively. In a model where a dominant effect was assumed, the genotype AA was coded as 0 while both Aa and aa were coded as 1. Accordingly, scores of 0 for both AA and Aa and 1 for aa were employed in a recessive model. In addition, under each genetic model the probabilities of *Y* being 1,2,3,4, and 5 were always 0.7,0.1,0.08,0.07, and 0.05, respectively. We set the covariance matrix Σ of U as

$${\text{Σ}}_{1}=\left(\begin{array}{ccc} 1 & 0.4 & 0.5\\ 0.4 & 1 & 0.8\\ 0.5 & 0.8 & 1 \end{array}\right)$$ (24)

to investigate the Type I error rate, and let Σ be

$${\text{Σ}}_{2}=\left(\begin{array}{ccc} 1 & \theta & 0.1\\ \theta & 1 & 0.1\\ 0.1 & 0.1 & 1 \end{array}\right)$$ (25)

to compare the statistical power under different alternatives, depending on the parameter *θ*, whose range was the set {−0.2,−0.1,0.1,0.2}.

Under the PO mechanism, the ordinal phenotype *Y* was related to the genotype *G* and covariate Z through the proportional odds model. Specifically, the distribution of *G* under different genetic models would remain the same as that under the ND mechanism, regardless of whether the HWE held. In this case, Z still followed the standard normal distribution, and *Y* was generated with five levels {1,2,3,4,5} using the proportional odds model

$$\begin{array}{l} \text{Pr}\left(Y\leq j\right)=\;[1+\text{exp}(-\alpha_{j}+Z+\beta_{1v}\text{I}\left(G=0\right)\\ \;{+\;\beta_{2v}\text{I}\left(G=1\right)+\beta_{3v}\text{I}\left(G=2\right)))]}^{-1}, \end{array}$$ (26)

where j=1,⋯,4, $\left(\alpha_{1},\alpha_{2},\alpha_{3},\alpha_{4}\right)=\left(0.85,1.39,1.99,2.94\right)$, $\left(\beta_{1v},\beta_{2v},\beta_{3v}\right)=v\times \left(0,0.15,0.3\right)$, v=0,5. It should be noted that Pr(Y≤5)=1. When v=0, the null hypothesis was true, and the derived simulation data were used to compare the Type I error rates for the three models of interest, *i.e.*, the lvm, probit, and logit models. When v=5, we explored the power of these three models under different alternatives.

As previously described, we considered 5×5×3+2×5×3+5×3×3+2×3×3=168 scenarios. For each simulation scenario, we generated 1000 datasets, each consisting of 300 subjects. P-values were calculated for each dataset using the three respective models. The nominal level of the tests was set to 0.05, and all simulations were performed using the R language (https://www.r-project.org/). The empirical Type I error rates and power estimates were calculated using the percentage of rejection in each scenario. The results are presented side-by-side in Tables 1–4. Note that the blanks (marked with —) in these four tables are a result of unavailability of the simulation data under the corresponding scenarios. The reason for this is that in these parameter setting conditions, the mean number for *G* amounting to 1 is three, which can easily lead to samples with all the *G* values being 0, such that none of the three considered models can be applied.

Table 1 presents the results for the ND mechanism when the HWE holds. The first five rows suggest that all three methods can control the Type I error rate at the nominal level of 0.05 under the three different genetic models. Furthermore, the remaining rows show that the lvm model enhances the statistical power over the probit and logit models. In some cases, the power gain can be as high as 0.07 to 0.1. For example, when θ=−0.2 and p=0.1, the empirical powers for the probit, logit, and lvm models are 0.514, 0.481, and 0.557 under the co-dominant genetic model, and are 0.503, 0.459, and 0.544 under the dominant genetic model, respectively. When the true genetic model is recessive and θ=−0.1 and p=0.3, the power estimates for the probit, logit, and lvm models are 0.126, 0.094, and 0.166, respectively.

**Table 1:** Type I error rates and power estimates under the ND mechanism (HWE holds)

*θ*	MAF	co-dominant			dominant			recessive		
		probit	logit	lvm	probit	logit	lvm	probit	logit	lvm
null	0.1	0.051	0.046	0.044	0.040	0.040	0.034	—	—	—
	0.2	0.049	0.049	0.040	0.053	0.054	0.048	0.049	0.058	0.069
	0.3	0.048	0.051	0.046	0.048	0.054	0.049	0.056	0.059	0.058
	0.4	0.049	0.052	0.041	0.050	0.042	0.041	0.046	0.046	0.045
	0.5	0.046	0.050	0.037	0.054	0.048	0.060	0.055	0.056	0.050
−0.2	0.1	0.514	0.481	0.557	0.503	0.459	0.544	—	—	—
	0.2	0.636	0.620	0.668	0.654	0.630	0.670	0.075	0.013	0.253
	0.3	0.713	0.685	0.736	0.657	0.654	0.668	0.309	0.246	0.390
	0.4	0.765	0.742	0.778	0.665	0.657	0.678	0.445	0.403	0.500
	0.5	0.757	0.747	0.768	0.605	0.607	0.617	0.580	0.556	0.610
−0.1	0.1	0.183	0.162	0.228	0.163	0.146	0.190	—	—	—
	0.2	0.250	0.237	0.267	0.218	0.209	0.233	0.031	0.006	0.138
	0.3	0.289	0.288	0.309	0.223	0.217	0.237	0.126	0.094	0.166
	0.4	0.240	0.234	0.251	0.233	0.239	0.244	0.158	0.128	0.195
	0.5	0.284	0.274	0.299	0.192	0.194	0.199	0.196	0.173	0.220
0.1	0.1	0.135	0.147	0.148	0.172	0.169	0.180	—	—	—
	0.2	0.195	0.197	0.202	0.162	0.161	0.169	0.083	0.094	0.128
	0.3	0.208	0.202	0.217	0.184	0.183	0.192	0.098	0.103	0.113
	0.4	0.210	0.196	0.217	0.174	0.159	0.190	0.131	0.132	0.138
	0.5	0.208	0.197	0.218	0.141	0.125	0.165	0.171	0.166	0.176
0.2	0.1	0.494	0.488	0.509	0.484	0.477	0.493	—	—	—
	0.2	0.590	0.586	0.598	0.585	0.573	0.595	0.237	0.248	0.269
	0.3	0.646	0.644	0.662	0.577	0.569	0.599	0.350	0.364	0.370
	0.4	0.675	0.665	0.685	0.546	0.533	0.573	0.446	0.448	0.446
	0.5	0.683	0.670	0.704	0.458	0.416	0.486	0.495	0.495	0.504

The results of the three methods under the ND mechanism when the HWE does not hold are presented in Table 2. We can observe that the proposed method achieves a greater power than the other two methods, even though the distribution of the genotype *G* does not satisfy the HWE conditions. Specifically, the power gain can be as high as 0.07 when θ=0.2 and the distribution of *G* is ${\text{P}}_{2}$.

**Table 2:** Type I error rates and power estimates under the ND mechanism (HWE does not hold)

*θ*	P	co-dominant			dominant			recessive		
		probit	logit	lvm	probit	logit	lvm	probit	logit	lvm
null	${\text{P}}_{1}$	0.060	0.061	0.055	0.049	0.039	0.052	0.054	0.054	0.049
	${\text{P}}_{2}$	0.042	0.050	0.043	0.043	0.037	0.060	0.059	0.056	0.050
	${\text{P}}_{3}$	0.050	0.045	0.053	0.040	0.032	0.046	0.059	0.058	0.059
−0.2	${\text{P}}_{1}$	0.745	0.741	0.754	0.556	0.551	0.564	0.653	0.627	0.662
	${\text{P}}_{2}$	0.775	0.759	0.788	0.570	0.581	0.586	0.613	0.587	0.642
	${\text{P}}_{3}$	0.684	0.667	0.700	0.583	0.575	0.589	0.351	0.299	0.428
−0.1	${\text{P}}_{1}$	0.248	0.247	0.266	0.194	0.195	0.198	0.243	0.232	0.256
	${\text{P}}_{2}$	0.302	0.286	0.315	0.197	0.194	0.199	0.220	0.205	0.244
	${\text{P}}_{3}$	0.238	0.224	0.253	0.192	0.192	0.198	0.161	0.120	0.198
0.1	${\text{P}}_{1}$	0.172	0.163	0.188	0.146	0.130	0.175	0.175	0.180	0.189
	${\text{P}}_{2}$	0.191	0.184	0.202	0.123	0.107	0.148	0.193	0.191	0.195
	${\text{P}}_{3}$	0.180	0.176	0.201	0.150	0.133	0.171	0.133	0.133	0.136
0.2	${\text{P}}_{1}$	0.638	0.620	0.669	0.479	0.440	0.523	0.564	0.542	0.579
	${\text{P}}_{2}$	0.669	0.665	0.687	0.465	0.429	0.499	0.535	0.519	0.545
	${\text{P}}_{3}$	0.615	0.605	0.633	0.442	0.411	0.471	0.383	0.399	0.401

The corresponding results when the data are generated under the PO mechanism and the HWE is assumed are displayed in Table 3. It follows that all three models can control the Type I error rates under the null hypothesis. Even though the data are generated using the proportional odds model, the lvm method still performs better than the other two methods in detecting an alternative hypothesis, and can achieve a power gain of up to 0.058 in some cases, such as the setting with p=0.3 for the recessive genetic model. It is worth noting that the advantage of the proposed lvm method is more obvious under the recessive model.

**Table 3:** Type I error rates and power estimates under the PO mechanism (HWE holds)

	MAF	co-dominant			dominant			recessive		
		probit	logit	lvm	probit	logit	lvm	probit	logit	lvm
null	0.1	0.047	0.039	0.056	0.051	0.051	0.062	—	—	—
	0.2	0.057	0.054	0.063	0.055	0.050	0.058	0.030	0.035	0.070
	0.3	0.050	0.049	0.054	0.051	0.049	0.054	0.042	0.039	0.057
	0.4	0.044	0.038	0.043	0.052	0.042	0.055	0.046	0.038	0.059
	0.5	0.048	0.044	0.055	0.059	0.056	0.063			
beta	0.1	0.580	0.563	0.598	0.519	0.512	0.534	—	—	—
	0.2	0.806	0.813	0.822	0.715	0.722	0.725	0.087	0.053	0.233
	0.3	0.897	0.900	0.901	0.763	0.776	0.775	0.291	0.254	0.349
	0.4	0.929	0.933	0.936	0.746	0.749	0.759	0.515	0.497	0.532
	0.5	0.929	0.932	0.929	0.682	0.694	0.697	0.647	0.646	0.655

The simulation results under the PO mechanism when the HWE does not hold are presented in Table 4. We observe that the advantage of the proposed lvm method is not as significant as that when the HWE holds, but the model is superior to the probit model in all scenarios. Moreover, the logit model has a slightly greater power under the co-dominant and dominant models, while the lvm method outperforms it in the recessive model.

**Table 4:** Type I error rates and power estimates under the PO mechanism (HWE does not hold)

	P	co-dominant			dominant			recessive		
		probit	logit	lvm	probit	logit	lvm	probit	logit	lvm
null	${\text{P}}_{1}$	0.050	0.054	0.063	0.054	0.053	0.059	0.055	0.051	0.057
	${\text{P}}_{2}$	0.052	0.051	0.053	0.048	0.046	0.050	0.058	0.059	0.058
	${\text{P}}_{3}$	0.054	0.057	0.060	0.056	0.060	0.061	0.056	0.053	0.068
beta	${\text{P}}_{1}$	0.968	0.975	0.972	0.589	0.624	0.599	0.755	0.774	0.765
	${\text{P}}_{2}$	0.936	0.937	0.926	0.595	0.608	0.602	0.698	0.706	0.713
	${\text{P}}_{3}$	0.737	0.761	0.756	0.605	0.632	0.620	0.329	0.293	0.375

From all of the four tables, it can be seen that the proposed method might have potential limitations in controlling Type I error rates in a few situations, while the power gains in almost all of simulation scenarios indeed indicate its efficiency for practical applications.

### Application to anticyclic citrullinated protein antibody data for rheumatoid arthritis study

It is well known that rheumatoid arthritis (RA) is significantly associated with some genetic variants (Carlton *et al.* 2005; Ruiz-Larrañaga *et al.* 2016). The anticyclic citrullinated protein antibody (anti-CCP) can be an auxiliary diagnosis indicator for RA, and the specificity of anti-CCP lies between 87.8% and 96.4% (Coenen *et al.* 2007). Besides, the genomic region of 6p21.33 has been reported to be associated with RA (Zhang *et al.* 2009; Zhang and Li 2015). The aim here is to check whether the single nucleotide polymorphisms (SNPs) in the 6p21.33 region are associated with anti-CCP, taking advantage of the proposed lvm test.

Note that there are a total of 45 SNPs in the region of 6p21.33 according to the Genetic Analysis Workshop 16 Data, all of which meet the quality control rule of the MAF being more than 5%, the missing rate being smaller than 15%, and the least genotype frequency being no less than five. The anti-CCP measure takes four values, 1, 2, 3, and 4, and the number of subjects who have these four values were 1195, 103, 66, and 698, respectively. The total number of subjects was 2062. Five principal components coordinated by applying the multi-dimensional scaling method (Li and Yu 2008) to the 12747 population structure information SNPs (Yu *et al.* 2008) were used to adjust for population stratification effects. Before conducting association analysis, we run chi-square tests to check whether HWE holds for each of these 45 SNPs in controls. The P-value results are summarized in Table 5. At a 0.05 level of significance, we can state that the HWE law holds for all SNPs in controls on the basis of their Bonferroni-corrected P-values. Then we apply the probit model, the logit model, and the lvm model to these 45 SNPs to test their association with anti-CCP in sequence. The results are presented in Table 5. It shows that after Bonferroni correction, the SNPs rs2246986, rs3093998, rs2071596, and rs2844509 were found to be significant under the probit and logit models, while the SNPs rs2516398, rs2844494, rs3130637, rs3093993, rs3095227, rs2259435, and rs2844509 were identified as significant using the lvm method. Though these two groups of SNPs overlap at only one SNP rs2844509, each of other SNPs found by the probit (logit) model is physically close to one or two SNPs found by the lvm model. For example, the SNP rs2246986 of the first group is 677 kb away from rs2516398 on one side and is 1212 kb away from rs2844494 on the other side, while both of these two SNPs rs2516398 and rs2844494 are included in the second group. In addition, the SNP rs3093998 (in the first group) is 2971 kb away from rs3130637 (in the second group). The distances are so short that it is reasonable to infer that the SNPs rs2246986, rs2516398, and rs2844494 contain similar information. So do another two SNPs rs3093998 and rs3130637. In short, for detecting the association between anti-CPP and the genomic region 6p21.33, the proposed lvm method is more powerful than the methods based on logit and probit models.

**Table 5:** P-values of 45 SNPs in the region of 6p21.33 for Genetic Analysis Workshop 16 Data

SNP ID	location	MAF	HWE test	probit	logit	lvm
rs6940467	31550116	0.3240	0.7354	0.0085	0.0065	0.1596
rs12660382	31551302	0.1076	0.8531	0.8904	0.9724	0.4209
rs2395488	31553888	0.1851	0.0639	0.0031	0.0036	0.1066
rs2248372	31554445	0.3515	0.6027	0.3260	0.3487	0.8352
rs2248373	31554525	0.3126	0.0696	0.6702	0.6757	0.6428
rs2248462	31554775	0.3329	0.0905	0.5906	0.6138	0.3037
rs2516513	31555567	0.2018	0.1526	0.4625	0.4408	0.3336
rs2516424	31556294	0.2037	0.0868	0.4737	0.4481	0.4048
rs2248617	31556512	0.3502	0.2845	0.3253	0.3478	0.9011
rs3828893	31556632	0.3508	0.8263	0.3865	0.4143	0.9098
rs3749946	31556841	0.0693	0.8614	0.8668	0.8451	0.6300
rs3099844	31556955	0.0677	0.3222	0.1556	0.1493	0.0249
rs2905722	31557306	0.1135	0.2584	0.2039	0.1443	0.0400
rs2523647	31557757	0.1355	0.9004	0.0119	0.0135	0.0020
rs2516509	31557973	0.2341	0.7044	0.0355	0.0284	0.0038
rs2523710	31558888	0.2003	0.0742	0.4901	0.4673	0.3584
rs2905747	31559455	0.1787	0.3843	0.0695	0.0644	0.0032
rs2523467	31565557	0.1996	0.2730	0.8405	0.9377	0.3975
rs2516415	31567721	0.3073	0.4062	0.8182	0.7386	0.8137
rs3130922	31569068	0.3172	0.0039	0.0097	0.0143	0.4089
rs3828903	31572718	0.3254	0.0015	0.0211	0.0148	0.0049
rs3828914	31573798	0.3179	0.0819	0.0524	0.0432	0.0152
rs2855812	31580699	0.2464	0.8426	0.0174	0.0239	0.3046
rs3134899	31581265	0.2357	0.5337	0.9991	0.9505	0.5082
rs2844498	31584833	0.4279	0.9148	0.9820	0.9221	0.4318
rs2246618	31586965	0.2886	0.0038	0.7829	0.8702	0.4483
rs2516400	31589084	0.3104	0.3859	0.7178	0.8215	0.3033
rs2516399	31589278	0.0886	0.4415	0.0051	0.0037	0.0158
rs2516398	31589505	0.3139	0.6824	0.0023	0.0022	0.0001
rs2246986	31590182	0.0807	0.8315	0.0010	0.0007	0.0106
rs2844494	31591394	0.3207	0.8615	0.0099	0.0101	0.0004
rs9267444	31591437	0.3363	0.6647	0.9317	0.8746	0.3115
rs3093998	31593153	0.3310	0.0233	0.0003	0.0007	0.0046
rs3130637	31596124	0.2390	0.0193	0.0137	0.0175	0.0004
rs3132454	31597623	0.3636	0.4028	0.8070	0.8352	0.3769
rs3093993	31598704	0.2388	0.0208	0.0139	0.0178	0.0004
rs3095227	31598979	0.2365	0.0477	0.0167	0.0210	0.0005
rs2259435	31604894	0.1800	0.2765	0.0094	0.0111	0.0002
rs3093983	31604904	0.1917	0.2858	0.4922	0.5327	0.1049
rs3130055	31605378	0.2765	0.3165	0.9600	0.9924	0.4228
rs3093978	31606476	0.1916	0.2337	0.3618	0.3981	0.0706
rs2734583	31613459	0.1109	0.0040	0.0329	0.0220	0.0059
rs2071596	31614670	0.1845	0.2263	0.00002	0.00002	0.0689
rs2516393	31614723	0.1911	0.2613	0.3905	0.4282	0.0776
rs2844509	31618903	0.2094	0.5601	0.0000	0.0000	0.0003

## Discussion

In this work, we have shown that the idea of treating a genotype variable as ordinal without assuming linearity can result in a more powerful and robust test, via introducing a joint multivariate normal distribution for the group of genotypes, traits, and covariates. Meanwhile, we have also demonstrated that the proposed lvm test can provide appropriate Type I error rates. The important strength of our method is that it does not make an assumption on the type of relationship between a phenotype and genotype; nor does it treat the genotype as a continuous variable. Rather, our approach only introduces a latent multivariate normal variable to characterize the relationship between the two, which is very reasonable, and generally considerably more useful.

Besides the simulations with respect to significance level of 0.05, we also conducted simulation studies with a lower significance level 0.005. The results are given in Supplementary Table S1. We found that the proposed method can reasonably control Type I error rates and achieve power gains at this lower significance level, similar to those results in Tables 1-4. It is worth mentioning that our proposed test model can also be applied to other situations where the outcome is continuous. In such a situation, we can still employ a joint multivariate normal distribution to model outcomes, genotypes, and covariates simultaneously. Even though the proposed method might have potential limitations in Type I error rate control in some situations, the power gains prove its efficiency in practical applications.

In population-based genetic association studies, hundreds of thousands of subjects are often enrolled to achieve optimal power. It is inevitable that there exists a population stratification effect in such large-scale studies. Not considering the effect of population stratification could lead to many false positive findings, and therefore adjusting for its effect represents the basis for conducting a genetic association analysis (Price *et al.* 2006; Li and Yu 2008). In this study, to characterize the influence of population stratification when investigating the relationships between ordinal traits and genotypes, we treat these effects as covariates in the proposed lvm method. The numerical results of our simulation studies and real data applications have demonstrated that the strategy is feasible and effective.
